# Factors influencing the implementation of clinical guidelines for health care professionals: A systematic meta-review

**DOI:** 10.1186/1472-6947-8-38

**Published:** 2008-09-12

**Authors:** Anneke L Francke, Marieke C Smit, Anke JE de Veer, Patriek Mistiaen

**Affiliations:** 1 NIVEL – Netherlands Institute for Health Services Research, PO Box 1568, 3500 BN Utrecht, The Netherlands

## Abstract

**Background:**

Nowadays more and more clinical guidelines for health care professionals are being developed. However, this does not automatically mean that these guidelines are actually implemented. The aim of this meta-review is twofold: firstly, to gain a better understanding of which factors affect the implementation of guidelines, and secondly, to provide insight into the "state-of-the-art" regarding research within this field.

**Methods:**

A search of five literature databases and one website was performed to find relevant existing systematic reviews or meta-reviews. Subsequently, a two-step inclusion process was conducted: (1) screening on the basis of references and abstracts and (2) screening based on full-text papers. After that, relevant data from the included reviews were extracted and the methodological quality of the reviews was assessed by using the Quality Assessment Checklist for Reviews.

**Results:**

Twelve systematic reviews met our inclusion criteria. No previous systematic meta-reviews meeting all our inclusion criteria were found. Two of the twelve reviews scored high on the checklist used, indicating only "minimal" or "minor flaws". The other ten reviews scored in the lowest of middle ranges, indicating "extensive" or "major" flaws.

A substantial proportion (although not all) of the reviews indicates that effective strategies often have multiple components and that the use of one single strategy, such as reminders only or an educational intervention, is less effective.

Besides, characteristics of the guidelines themselves affect actual use. For instance, guidelines that are easy to understand, can easily be tried out, and do not require specific resources, have a greater chance of implementation.

In addition, characteristics of professionals – e.g., awareness of the existence of the guideline and familiarity with its content – likewise affect implementation.

Furthermore, patient characteristics appear to exert influence: for instance, co-morbidity reduces the chance that guidelines are followed.

Finally, environmental characteristics may influence guideline implementation. For example, a lack of support from peers or superiors, as well as insufficient staff and time, appear to be the main impediments.

**Conclusion:**

Existing reviews describe various factors that influence whether guidelines are actually used. However, the evidence base is still thin, and future sound research – for instance comparing combinations of implementation strategies versus single strategies – is needed.

## Background

Evidence based medicine (EBM) is the conscientious, explicit, and judicious use of current best evidence in making decisions about the care of individual patients [1]. EBM is increasingly embraced by professional associations, as well as by international health care organizations, such as the WHO [2,3]. Clinical guidelines, in the sense of scientifically developed statements to assist practitioner and patient decision-making about appropriate care for specific clinical conditions, may be important instruments to shape evidence based medicine [1,4]. Professionals can use guidelines for decision-making at the bedside of individual patients. The guidelines may provide instructions on which diagnostic or screening tests or interventions to be used [5]. Guidelines are also increasingly regarded as being an indispensable part of professional quality systems [6]. These may, for instance, involve continuing professional education, peer review and audit procedures [7]. Accordingly, in many ways, clinical guidelines help practitioners to improve their professional practice and the quality of care and – subsequently – patients' outcomes.

In addition, guidelines may empower patients to make more informed health care choices [8].

Although the development of guidelines for medical staff, nursing staff and/or other health care professionals has gained momentum in recent years, this does not necessarily mean that the recommendations described in the guidelines are actually followed [4,9-12]. For instance, Grol et al. [13] concluded in an observational study on ten Dutch guidelines, that guideline recommendations were followed by GPs in an average of 61% of the relevant decisions. In addition, Bauer [14] analysed 41 studies on the implementation of clinical guidelines in the field of mental health care, including depression, schizophrenia and addiction. Guideline adherence was found in 27% of the cross-sectional and pre-post studies and in 67% of the controlled trials under review. Several of these studies showed that after the cessation of specific implementation strategies, adherence rates returned to baseline levels.

This article primarily aims to give insight into factors that negatively or positively influence the implementation of clinical guidelines. Since many guidelines are currently being developed, but may not always be successfully implemented, this is a relevant topic.

A second aim of this systematic meta-review is to shed light on the "state-of-the-art" regarding research within this field. Meta-reviews in particular are appropriate for describing whether the current evidence base is complete or incomplete, since evidence from relevant previous systematic reviews or meta-reviews is synthesized. The reason for including only systematic reviews or meta-reviews is because this kind of research generally provides more evidence than separate empirical studies.

This systematic meta-review addresses the following research question:

- What evidence exists regarding factors that influence the implementation of clinical guidelines either negatively or positively?

In this article, the term "implementation of" guidelines is sometimes replaced by terms like "use of", "adherence to" or "compliance with" (depending on the terminology used in the publications we are referring to).

## Methods

### Searches in databases

To find relevant publications, we developed a search strategy in cooperation with an experienced librarian. The search strategy was developed first for Pubmed, to be adapted later to search other databases (with no time period limitation). Table 1 displays the search strategy for Pubmed. This strategy was adapted for the other literature databases cited in Table 2. All searches were executed in November 2006.

**Table 1:** Search strategy in Pubmed

#1 Search **review [TIAB]**
#2 Search **review [pt] OR meta-analysis [pt]**
#3 Search **meta-analysis [TIAB]**
#4 Search **"Practice Guidelines" [MAJR] OR "Critical Pathways" [MAJR]**
#5 Search **Practice Guideline [pt]**
#6 Search guideline* [ti] OR protocol* [ti]
#7 Search develop* [ti] OR implement* [ti]
#8 Search **"Guideline Adherence" [MAJR] OR "Organizational Culture" [MAJR] OR "Outcome and Process Assessment (Health Care)" [MAJR] OR "Attitude of Health Personnel" [MAJR] OR "Health Plan Implementation" [MAJR] OR "Information Dissemination" [MAJR]**
#9 Search **#1 OR #2 OR #3**
#10 Search **#4 OR #5 OR #6**
#11 Search **#7 OR #8**
#12 Search **#9 AND #10 AND #11**
#13 Search **letter [pt] OR comment [pt] OR editorial [pt]**
#14 Search **#12 NOT #13**
#15 Search **#12 NOT #13 **Limits: **only items with abstracts**

(((review [TIAB] OR review [pt] OR meta-analysis [pt] OR meta-analysis [TIAB]) AND ("Practice Guidelines" [MAJR] OR "Critical Pathways" [MAJR] OR Practice Guideline [pt] OR guideline* [ti] OR protocol* [ti]) AND (develop* [ti] OR implement* [ti] OR "Guideline Adherence" [MAJR] OR "Organizational Culture" [MAJR] OR "Outcome and Process Assessment (Health Care)" [MAJR] OR "Attitude of Health Personnel" [MAJR] OR "Health Plan Implementation" [MAJR] OR "Information Dissemination" [MAJR])) NOT (letter [pt] OR comment [pt] OR editorial [pt])) AND (has abstract[text])

**Table 2:** Literature data bases and number of references (sometimes overlapping between data bases)

**Data base**	**References**
Pubmed	466
Cinahl	188
Cochrane library (excluding clinical trials register)	222
Embase	443
NIVEL catalogues	40
GIN-website	0

The references resulting from the searches were entered in Reference Manager and within this program duplicates were removed. Without duplicates, 885 references remained.

### Inclusion criteria

In order to assess whether the references found were indeed relevant, we formulated the following criteria concerning types of studies, target groups and variables.

1. Studies: only systematic reviews or meta-reviews were eligible for inclusion. We considered a review or meta-review to be systematic if at least two of the following three criteria were satisfied: (a) search terms are presented; (b) Pubmed/Medline, at least, has been searched; (c) the methodological quality of the included studies has been assessed by the reviewer(s).

2. Target groups: the guidelines mentioned in the reviews or meta-reviews should be aimed at medical staff, nurses or other professionals in health care

3. Variables: the systematic reviews or meta-reviews should discuss factors that influence guideline implementation either positively or negatively.

### Exclusion criteria

Reviews or meta-reviews that *exclusively *focussed on consensus procedures or consensus-based guidelines were not included (e.g. [15-17]). By consensus-based guidelines we mean guidelines that have been developed exclusively on the basis of consensus procedures, without a systematic analysis of relevant scientific literature. We decided to rule out (meta-)reviews that focussed exclusively on consensus-based guidelines, because we assume that clinical guidelines should be based as much as possible on scientific evidence [18].

Additionally, reviews or meta-reviews that do not differentiate in their conclusions between clinical guidelines and other professional interventions, such as continuing education or comprehensive quality programs, were excluded [19-23].

Next, reviews or meta-reviews were ruled out if their findings are not only based on research publications, but also on descriptive, narrative or theoretical articles (e.g. [3,11,24-40]).

*No *exclusion criteria were applied regarding language or search period.

### Inclusion process

The inclusion process took place in the following steps.

#### Step 1 – screening of titles and abstracts

Titles and abstracts of the references found (n = 885) were screened independently by a first and second meta-reviewer (ALF and MCS), to check whether these publications satisfied the inclusion criteria. In this phase the two reviewers agreed in virtually 100% of the cases. For the references selected by both reviewers as well as for the references selected by only one reviewer (a total of 84), we tried to track down or download the full text.

#### Step 2 – screening based on full texts

Next, the full texts were assessed independently by the first and second meta-reviewer (ALF and MCS) using the inclusion criteria cited.

In this phase, we conducted a manual search in the reference lists of the full text papers. Subsequently, we were able to add another 24 potentially relevant references. The full texts of these additional references were studied as well, which brings the total number of full texts examined to 108.

When the first and the second reviewer did not agree in this phase on inclusion or exclusion (initial disagreement existed in this phase for only two publications), agreement was reached on the basis of discussion between the two reviewers.

Ultimately, twelve review studies, all displayed in Additional file 1, appeared to be eligible for inclusion. One of these reviews was described in two publications [41,42].

Table 3 shows the steps of the inclusion process:

**Table 3:** Flow diagram of the searches and inclusion process

**Searches:**	
a total of 885 potentially relevant references	
↓	
**Inclusion step 1:**	
Screening based on titles and abstracts: 84 publications selected	
↓	
**Inclusion step 2:** Screening based on 108 full texts (84 full texts derived from previous step and 24 additional titles derived from reference tracking) ↓ ↓ ↓ ↓ ↓ ↓ ↓	Reasons for **exclusion **were: * *not *meeting inclusion criterion 1 (systematic review or meta-review): n = 48 * *not *meeting inclusion criterion 2 (guideline target-groups concern medical staff or other health professionals): n = 7 * *not *meeting inclusion criterion 3 (focus on factors influencing guideline implementation): n = 17 * meeting 1 or more *ex*clusion criteria (exclusively dealing with consensus-based guidelines, not clearly differentiating between guidelines and other professional interventions, or not exclusively based on research publications): n = 23
**12 review studies included **(described in a total of **13 **publications)	

### Methodological assessment

The methodological quality of the twelve included studies was then assessed by two meta-reviewers (ALF and PM) independently, with the Quality Assessment Checklist for Reviews [43,44] (see Additional file 2). This checklist is used quite frequently for assessing systematic reviews methodologically [45,46]. The checklist is one of the few found for which psychometric properties had been documented [47] and which had been shown to meet several important criteria, such as inter observer reliability and coverage of the items in the QUORUM statement for reporting systematic reviews [48]. The overall scores on this checklist range from "extensive flaws" (score 1 or 2), to "major flaws" (score 3 or 4), "minor flaws" (score 5 or 6) and "minimal flaws" (score 7).

When the overall score of the meta-reviewers' assessments for a particular review study differed, the average of the mutual overall score was calculated. In the case of large differences (> 1 point) in mutual overall scores, agreement was reached on the basis of discussion between the meta-reviewers. The scores of the appraisal are shown in Table 4. The checklist and an explanation of its use are contained in Additional file 2.

**Table 4:** Outcomes of methodological assessment on the Quality Assessment Checklist for Reviews

**Reference of the review**	**Total score on checklist**
Bauer [14]	3
Cabana et al. [49]	4.5
Davies et al. [58]	3
Davis & Taylor-Vaisey [57]	1.5
Grilli omas [53]	3.5
Grimshaw et al. [41,42]	7
Gross & Pujat [54]	3
Sachs [51]	4
Saillour-Glenisson & Michel [52]	2.5
Simpson et al. [50]	3.5
Thomas et al. [56]	5.5
Tooher et al. [55]	3.5

### Data analysis and synthesis

The data from the twelve included reviews are presented descriptively following the structure of Additional file 1. Factors that influence, or could influence, the implementation of clinical guidelines are classified in the following categories:

* characteristics of the guidelines;

* characteristics of the implementation strategies;

* characteristics of professionals;

* characteristics of patients;

* characteristics of the environment.

This categorization was chosen because of its compatibility with classifications that are used in some of the assessed studies, such as that of Cabana et al. [49] and Simpson et al. [50].

Data extraction was performed by the first reviewer (ALF) and the data were subsequently checked by another reviewer (AJEDeV or PM).

The reviews included determined whether a factor was described as having a positive or a negative influence on guideline implementation. Because of the large variety of factors described and methods used, no quantitative pooling was performed across the reviews. Moreover, pooling was not possible since the large majority of the reviews studied did not provide numbers, e.g. in the form of effect sizes. Conclusions for the meta-review were therefore based on the conclusions and results presented in the reviews.

In the Results section as well as in the Conclusion section our findings are discussed in relation to the methodological quality of these review studies.

## Results

### General description of the reviews and the guidelines

The twelve included studies are all systematic reviews; no previous systematic *meta*-reviews were found that completely satisfied our inclusion criteria.

Nearly all twelve review studies concerned English language studies only, although this was not always the result of an explicitly stated exclusion criterion regarding language. Two reviews did however also include non-English studies: the German language review by Sachs [51] and the French language review by Saillour-Glenisson and Michel [52], which also included publications in German and French respectively. The aforementioned study by Sachs [51] is also the most recent review included. The review by Grilli and Lomas [53] is the oldest.

The reviews included had varying objectives: some aimed to map the success and failure factors or the most effective implementation strategies; others focussed primarily on charting the effects of clinical guidelines (see aims in Additional file 1, column 1). In these cases, establishing which factors had influenced successful implementation was not the primary focus, although the reviews provided relevant information in this regard.

There is also limited overlap in the subjects to which the guidelines relate. For instance, the guidelines in the review by Gross and Pujat [54] had a rather narrow focus and described recommendations for the use of antibiotics. Likewise, the reviews by Simpson et al. [50] and Tooher et al. [55] dealt with a specific topic, viz. the treatment of pneumonia and pressure ulcers respectively. Other reviews had a broader focus and concerned a number of guidelines on preventive or curative treatments and various diseases (see Additional file 1).

In most instances, the main target groups of the guidelines appear to be physicians. The majority of review studies do not explicitly state who the main target groups are, or whether the clinical guidelines are mono-disciplinary or multi-disciplinary (see Additional file 1, column 1). The descriptions and results often show only indirectly that physicians were the main target group. By contrast, the reviews of Sachs [51] and Thomas et al. [56] made it very clear that they were aiming at other target groups: Sachs [51] concentrated on guidelines for nursing staff and Thomas et al. [56] dealt with guidelines for nursing staff, midwives and/or other allied health care professionals.

### Methodological characteristics of the reviews

As stated earlier, for the structured methodological assessment of the twelve reviews we used the Quality Assessment Checklist for Reviews of Oxman and Guyatt (See Additional file 2).

Two reviews received a high score based on the Quality Assessment Checklist for Reviews: the review by Thomas et al. [56] received a score of 5.5, and that of Grimshaw et al. [41,42] scored a 7. These scores reflect "minor flaws" and "minimal flaws" respectively on the checklist. The high scores are related to the fact that the searches by these reviewers were extensive and well documented. For instance, they described their search strategy; their search for evidence was comprehensive; and they applied clear inclusion criteria. They also took measures to prevent selection bias, by involving more than one reviewer in the selection process. In addition, in these two reviews the methodological quality of the included studies was systematically assessed [41,42,56].

The remaining ten reviews received a score of between 1.5 and 4.5, which relate to the lowest or middle ranges of the checklist, indicating "extensive" to "major" flaws (for the interpretation of these scores also see Additional file 2in relation to Table 4).

Additional file 1 (column 1) shows that most reviews included quantitative studies with comparative designs (RCTs, CCTs, pre-test post-test studies), enabling the reviewers to make statements about the differences between (the effects of) implementation strategies. This is not the case with the review by Cabana et al. [49] which included only surveys and qualitative studies, in order to disclose the barriers to the implementation of clinical guidelines. The reviews of Saillour-Glenisson and Michel [52] are also slightly different; they included both quantitative and qualitative studies to reveal such barriers. Grilli and Lomas [53] included quantitative studies, but in the studies with pre-measurements and post-measurements they only looked at the post-measurements, in order to be able to write about adherence to clinical guidelines.

All twelve reviews not only searched Pubmed/Medline but also studied other sources, and they often used explicit inclusion criteria (see Additional file 1, column 2). Remarkably, most reviews pay no attention to preventing selection bias or to methodological assessment. In addition, information on which methods were used to synthesize study results and to reach conclusions was not, or was only partially provided in the reviews.

### Factors influencing implementation: characteristics of the guidelines

The most frequently described guideline characteristic concerns complexity. Guidelines that are easy to understand, can easily be tried out, and do not require specific resources have a greater chance of being used (Davis and Taylor-Vaisey [57], Grilli and Lomas [53], Saillour-Glenisson & Michel [52] and Simpson et al. [50])

Other influential guideline characteristics are also described, although not so frequently as the factor "complexity of the guideline". For instance, the review of Saillour-Glenisson & Michel [52] concluded that adherence to evidence based guidelines appears to be higher than is the case for guidelines lacking a clear scientific base. In addition, Sachs [51] concluded that when guidelines are developed by the target group (in that case nurses) and experts, this enhances the chance of successful implementation. However, Davies et al. [58] maintain that the findings are contradictory with regard to whether guidelines that are developed by end users (amongst others) are more often used.

For other – not frequently described – influential guideline characteristics, see Additional file 1 (column 3).

In interpreting the results, it should however be taken into account that all reviews describing the influence of guideline characteristics [49-53,57] have a relatively low methodological score (4.5 or lower) on the Quality Assessment Checklist for Reviews. This implies a high likelihood of "extensive" or "major" flaws in the results and conclusions.

### Characteristics of the implementation strategies

Almost all reviews examined the characteristics of the implementation strategies in relation to the use of the guideline (see Additional file 1).

In the review of Grimshaw et al. [41,42], 235 studies with comparative designs (RCTs, CCTs etc.) on implementation strategies regarding guidelines with a broad variety of topics were analysed. As already described, the Grimshaw review received a high methodological score, namely 7, on the checklist used. This review [41,42] described the effects of several implementation strategies. For instance, the authors investigated the effects of combined educational materials and meetings as well as combinations of educational materials and audit and feedback. Other combinations were also investigated (see Additional file 1). For most combinations as well as for most single strategies, Grimshaw et al. [41,42] found some effects. However, they also stated that the effects are often modest and the evidence base sparse. Although these authors described some studies that found more effects of combined strategies than of single ones, one of their main conclusions was that there is no evidence that multi-faceted strategies are more effective than single ones. In addition, they concluded that there was no significant relationship between the number of components of multi-faceted strategies and the effects measured.

As stated earlier, Thomas et al. [56] were the authors of the other review with a high methodological score (5.5) on the Quality Assessment Checklist for Reviews. These researchers [56] only focussed on studies regarding guidelines for nurses or allied health professionals and only described three studies relevant to the questions in our meta-review. These three studies were all compromised by a small sample size or "unit of analysis" errors, and the conclusion was that insufficient evidence existed about the effectiveness of different implementation strategies with regard to guidelines aimed at nurses or allied health professionals.

Some reviews with lower methodological scores (scoring 4.5 or lower on the checklist) did not point in the same direction as Grimshaw et al. [41,42]. For instance, Bauer et al. [14] concluded that multi-faceted and intensive strategies, involving system redesign or additional resources (e.g. regarding additional consultation or case management) seem to be most successful in improving adherence to mental health guidelines. Davis and Taylor-Vaisey [57] focussed on guidelines with a variety of subjects and concluded that single strategies (e.g. reminder systems) may be effective, but strategies involving two or more interventions often appear to have greater impact. Besides, Tooher [55], reaches the conclusion that with regard to guidelines on pressure ulcers, the more comprehensive the implementation strategies are (that is, the greater the variety and breadth of the strategies) the more effective their implementation seems to be in the long term.

Comparable conclusions on the value of multi-faceted strategies versus single strategies are presented by Gross and Pujat [54] and Sachs [51].

For single strategies only, there appears to be insufficient evidence to reach conclusions about the *relative *effectiveness of different implementation strategies in different contexts or circumstances (Grimshaw et al. [41,42] and Davies et al. [58]) However, according to the review of Davies et al. [58], strategies requiring active professional participation, and strategies that are closely related to clinical decision- making are more likely to lead to successful implementation In other words: strategies that are closer to the end user and more integrated into the process of health care delivery appear to be most successful.

### Characteristics of professionals

Six of the twelve reviews paid attention to certain characteristics of professionals in relation to the implementation of clinical guidelines [49-52,57,58]. All these reviews received a methodological score of 4.5 or lower, indicating a high chance of "extensive" or "major" flaws.

The review of Cabana et al. [49] was the most detailed, and described characteristics of physicians in relation to the adoption of clinical guidelines on a number of issues (see Additional file 1, column 3). One of the conclusions of this review is that a lack of awareness, limited familiarity and a lack of agreement with guidelines are the main barriers to guideline adoption. The results of the Cabana review [49] point largely in the same direction as the reviews of Saillour-Glenisson and Michel [52] and Simpson et al. [50]. Also these reviews conclude that the main barrier can be found in the simple fact that physicians are sometimes not aware of the existence of particular guidelines. In addition, three reviews also mention age and/or experience as determinants: young professionals or less experienced ones would be more inclined to use guidelines than older, experienced professionals [50,52,57]. Other characteristics of professionals that influence implementation are listed in Additional file 1 (column 3).

### Characteristics of patients

Four reviews with a methodological score of 4.5 or lower on the Quality Assessment Checklist for Reviews described the influence of a rather limited number of patient characteristics [49,50,52,57]. Cabana et al. [49] concluded that patient-related characteristics may include the fact that some patients perceive no need for guideline recommendations or may even resist them. Saillour-Glenisson and Michel [52] also described resistance of patients towards the recommendations as a factor negatively affecting the adoption of clinical guidelines.

In addition, Davis and Taylor-Vaisey [57] refer to patients with co-morbidity: in the case of such patients the chance is greater that professionals do not adhere to guidelines. Additional relevant patient characteristics are listed in Additional file 1.

### Environmental characteristics

Six reviews, again all with a methodological score of 4.5 or lower on the Quality Assessment Checklist for Reviews, studied environmental characteristics [49-52,55,57].

Limited time and personnel resources as well as work pressure [49-52] are rather frequently cited environmental characteristics said to negatively influence guideline implementation. A negative attitude or limited support from "peers" or superiors also has a negative influence [51,52,55,57]. Other environmental characteristics are stated in Additional file 1.

## Discussion

### Factors influencing the implementation of guidelines

The most frequently investigated guideline characteristic concerns "complexity". Several of the systematic reviews included in our meta-review indicated that when a guideline can be relatively easily understood and tried out, the chance is greater that the guideline will be used.

It is important therefore for guideline developers to take into account the complexity of the guidelines. Particularly for developers of multi-disciplinary guidelines directed at several target groups with varying educational levels and backgrounds (e.g. physicians, nurses, patients), it is a challenge to describe recommendations that are understandable and usable for all target groups.

The finding in the Sachs review [51], that involving the targeted professionals already in the development phase enhances the chance of successful implementation, may be relevant for guideline developers as well. Prominent groups like the WHO Advisory Committee on Health Research [27] and the AGREE Collaboration [59]also recommend that groups that develop guidelines should be broadly composed and include all relevant health professionals. In addition, involvement of the target group may imply that the guideline is first being tested in practice before large-scale implementation takes place [59].

Still, Davies et al. [58] assert that findings are not always unanimous with regard to whether guidelines that are developed by end users (amongst others) are more often used. Future research will have to provide more insight into this issue.

Our meta-review also describes specific influential characteristics of professionals. Implementers of guidelines and policy makers in particular should take into account that implementation may be hampered by the simple fact that professionals are often unaware that the guidelines exist, or are not familiar with their content [49]. Clearly, it is not sufficient to merely disseminate a guideline. Targeted implementation interventions – in which professionals themselves are preferably directly and actively involved – should take place to create awareness. Examples of such targeted interventions may be combinations of (web-based, written or face-to-face) practical recommendations, educational material, and educational meetings (see for instance the Sachs article [51]).

Characteristics of patients, too, appear to exert influence: for instance, co-morbidity in a patient appears to reduce the chance that guidelines are followed [57]. Professionals, presumably, assume that guidelines are based on a general clinical picture and are insufficiently tailored to the often complex care needs of patients with co-morbidity. For instance, Tinetti et al. [60] and Durso [61] therefore argue for greater attention among guideline developers to the specific needs of patients with co-morbidity. To improve guideline implementation, these authors recommend that guidelines should also provide guidance for interventions in patients with multiple conditions as well as information on risks of specific interventions in these patients.

In addition, environmental characteristics influence the implementation of guidelines. For example, support by peers or superiors in following the guidelines, and sufficient staff and time appear to be important for guideline implementation [49].

However, with respect to environmental characteristics, and also regarding patients' and professionals' characteristics, existing systematic reviews lack methodological rigour, and underlying primary research often focuses on rather heterogeneous guideline subjects and target groups. This hampers evidence-based conclusions. Future sound methodological research regarding these kinds of characteristics is therefore recommended.

More research is already being performed regarding the category "characteristics of the implementation strategies". Almost all included reviews investigated the influence of certain implementation strategies (see Additional file 1), which provides relevant information, particularly for guideline implementers. Most of the reviews indicate that effective implementation strategies often have multiple components and that the use of one single strategy, such as reminders only or one educational intervention, is less effective than a combination of strategies. However, interpretation is hampered by the fact that the high-quality Grimshaw review published in 2004 and 2006 [41,42] does not show a correlation between the number of components in implementation strategies and their effectiveness.

### Explanations for contradictory results

The contradictory results found for "characteristics of the implementation strategies" may be partially explained by limited overlap in target groups and guideline subjects. The Grimshaw review included a large number of primary studies (n = 235) on guidelines with varying target groups and topics, while other relevant reviews involved fewer studies and had a narrower focus, e.g. on guidelines for mental health care [14], guidelines for pressure ulcers [55] or guidelines for nursing practice [51].

Another (partial) explanation may be that Grimshaw et al. [41,42] were more rigorous in their analysis of primary studies than the other reviewers. Although Grimshaw and colleagues did not conduct a formal meta-analysis (because of the large heterogeneity of studies), they did take effect sizes into account.

However, a methodologically strong review by Wensing et al. [23] also contradicts the conclusion of Grimshaw et al. [41,42]. Wensing and colleagues reviewed studies on a rather broad range of professional, educational and quality interventions (which was why the Wensing review did not completely match our inclusion criteria, and was not discussed in previous sections). Wensing et al. concluded that combined implementation strategies with many different aspects are, in general, the most effective. Furthermore, findings of two other methodologically sound meta-reviews [20,62] are relevant in this regard. These previous meta-reviews were likewise excluded from our meta-review since they focussed on a broad range of professional or educational interventions, and not specifically on clinical guidelines. Yet these meta-reviews equally concluded that successful implementation strategies are often multi-faceted.

## Conclusion

Our main conclusion is therefore that multiple strategies for implementing guidelines appear to be more effective than single ones. In the introduction we stated that guidelines are increasingly considered to be part of comprehensive quality systems [6], often combining guidelines with educational interventions, audits and other actions for improvement. The conclusion, that multiple strategies seem to be most effective, fits with the comprehensive character of today's quality systems.

However, guideline researchers in particular, should be aware that well-constructed empirical research looking into various implementation strategies is still needed in this area [11]. This will enable us to make more definitive statements about the effectiveness of multi-faceted strategies compared to specific single strategies in implementing clinical guidelines.

### Methodological considerations

At the end of this paper, we will discuss some methodological issues concerning our own meta-review. Ten out of the twelve reviews described in this meta-review scored in the low or middle ranges of the Quality Assessment Checklist for Reviews, which indicates "major" or "extensive" flaws (see Additional file 2). Initially, we considered excluding reviews with low or middle range methodological scores, in order to reduce the risk of bias. However, this would have left us with only a very limited number of reviews to be included. We want to offer a complete picture of existing research: this is why we decided after all to include all relevant systematic review studies meeting our inclusion criteria. Regarding this decision, it should also be taken into account that strict criteria for the methods of systematic reviews have only become common practice in recent years, brought about – for instance – by the Cochrane Collaboration. This makes it understandable that less recent review studies in particular do not fully satisfy these criteria or scarcely mention the review methods they have used. Consequently, reviews with a relatively low methodological score do not automatically show distorted results and may sometimes provide valuable insights.

A limitation of this meta-review is that our search for potentially eligible publications ceased in November 2006. Analyzing and synthesizing the results of previous reviews are time-consuming procedures, and therefore a time span between the searches and the submission to a journal cannot be avoided. However, in order to be sure that we did not miss relevant information from very recent papers, we performed an additional search in Pubmed from November 2006 to February 2008, just before submission. This resulted in two additional publications meeting the inclusion criteria; one concerned successful characteristics of implementation strategies for guidelines in obstetrics [63], while the other focussed on characteristics of strategies for implementing psychiatric guidelines [64]. Neither of these publications presents results that might have altered our conclusions. In line with our above-outlined conclusion on implementation strategies, both recent papers concluded that multi-faceted strategies are generally the most effective.

## Competing interests

The authors declare that they have no competing interests.

## Authors' contributions

ALF: conception and design of the meta-review; searches and selection of literature; analysis and synthesis of data from the included literature; drafting the manuscript. MCS: conception and design of the meta-review; searches and selection of literature; revising the first draft of the manuscript. AJEdeV: conception and design of the meta-review; analysis and synthesis of data from the included literature; revising the first draft of the manuscript. PM: conception and design of the meta-review; analysis and synthesis of data from the included literature; revising the first draft of the manuscript.

## Pre-publication history

The pre-publication history for this paper can be accessed here:



## Supplementary Material

Additional file 1 Table 5 – Data extracted from the included reviews, for as far relevant for our meta-review.
The data provided represents summarized data from the reviews included in our meta-review. Click here for file

Additional file 2 Annex.
This file displays a checklist for assessing methodological quality. Click here for file
