# Supplementary material for: Factors influencing the implementation of clinical guidelines for health care professionals: A systematic meta-review
Source: BMC Med Inform Decis Mak. 2008 Sep 12;8:38. doi: 10.1186/1472-6947-8-38 (PMC2551591; doi:10.1186/1472-6947-8-38)
Supplement: Additional file 1 — Table 5 – Data extracted from the included reviews, for as far relevant for our meta-review. The data provided represents summarized data from the reviews included in our meta-review. [file 1472-6947-8-38-S1.doc]

Table 5 - Data extracted from the included reviews, for as far relevant for our meta-review

| **Column 1**  **References, aims and short description** | **Column 2**  **Methodological characteristics** | **Column 3**  **Results** | **Column 4**  **Conclusions** |
| --- | --- | --- | --- |
| **Bauer [14]**  **Aim:** to review peer reviewed reports providing quantitative information on rates of adherence to mental health guidelines  **Studies included:** 41 English language studies reporting adherence rates expressed as % of patients who received, or % of providers who delivered/reported guideline adherent care.  **Guideline topics:** recommendations for mental health care, by professional bodies or regulatory agencies (no local guidelines).  **Guideline target groups:** varying | **Design of studies included:** cross sectional, pre-test post-test studies or CCTs  **Searches in:** Medline, Cochrane controlled clinical trials database (to 2000). Additional literature was found by consulting colleagues and bibliographies  **Search strategy described:** yes  **Inclusion criteria explicitly reported:** yes  **Interventions to reduce selection bias:** no  **Methodological assessment included studies:** no  **Adequate methods used to combine studies and reach conclusions reported:** partially | **Characteristics of guidelines: ---**  **Characteristics of implementation strategies:**  - Multi-faceted and intensive strategies, involving system redesign or additional resources ( e.g. regarding additional specialty consultation or additional case management) seem to be relatively successful  - Academic detailing (which is as a process by which a health educator visits a professional to provide a short educational intervention on a specific topic) or other educational strategies or /feedback are – when they are not combined with other interventions - not sufficient to improve adherence to guidelines  **Professionals’ characteristics:** ---  **Patient characteristics:** ---  **Environmental characteristics:** --- | Successful interventions are typically complex and multi-modal, and should be maintained to sustain the goals |
| **Cabana et al. [49]**  **Aim:** to review barriers to physician adherence to guidelines **Studies included:** 76 English language studies on adherence of guidelines and recommendations, examining at least 1 barrier to adherence.  **Guideline topics:**a variety of topics, e.g. all kinds of preventive and curative treatments  **Guideline target groups:** (mainly) physicians | **Design of studies included:** surveys and qualitative studies  **Searches in:** Medline, Eric and Healthstar (Jan 1966 to Jan 1998), references of bibliographies, textbooks and references supplied by experts  **Search strategy described:** yes  **Inclusion criteria explicitly reported:** yes  **Interventions to reduce selection bias:** 1 researcher screened all titles, then 2 researchers independently reviewed full texts to include/ exclude studies  **Methodological assessment included studies:** no  **Adequate methods used to combine studies and reach conclusions reported:** yes | **Characteristics of guidelines:**  **- in the relevant studies a**t least 10% of the respondents described guidelines as not easy to use, not convenient, cumbersome or confusing.  **Characteristics of implementation strategies:---**  **Professionals’ characteristics:**  - Lack of awareness: in 78% of the relevant surveys, at least 10% of the respondents were not aware of the guideline  - Lack of familiarity*:* in 90% of the relevant surveys, at least 10% of the respondents was not familiar with guideline recommendations  - Lack of agreement: in 62% of the, at least 10% of the respondents reported lack of agreement with a specific guideline  **- Lack of self efficacy: i**n 79% of the surveys, at least 10% of the respondents reported a lack of self efficacy  **- Lack of outcome expectancy: i**n 88% relevant surveys (7 of 8), at least 10% of the respondents reported a lack of outcome expectancy  **- Inertia of previous practice: i**n all relevant surveys more than 10% of the respondents reported inertia of previous practice as a barrier  **Patient characteristics:**  In all relevant surveys at least 10% of the respondents indicated patient characteristics as barriers (e.g. resistances of patients or patients may perceive recommendation as offensive or embarrassing)  **Environmental characteristics:**  - In all relevant surveys at least 10% indicated that environmental characteristics (e.g. insufficient staff or consultant support, lack of material, poor reimbursement, increased costs) influenced adherence  The importance of **patient characteristics** or time constraints (an **environmental characteristic**) are also emphasised by 4 of the 5 qualitative studies | There are many barriers for guideline adherence of physicians. However, results may not always be generalizable, since barriers in one setting may not be present in another. |
| **Davies et al. [58]**  **Aim**: to examine (a) the evidence on whether guidelines can change behaviour of professionals, (b) how guidelines can best be introduced in practice, (c) characteristics of high quality guidelines and (d) how purchasers might use guidelines.  **Studies included:** 91 English language studies  **Guideline topics:** varying, topics concerned clinical care, preventive care or prescribing, laboratory or radiological investigations in a variety of settings.  **Guideline target groups:** (primarily) physicians | **Design of studies included:** RCTs, randomised crossover-trials, balanced incomplete block designs, controlled before-and-after studies, interrupted time series  **Searches in:** Medline, DHSS-DATA, Embase and SIGLE (all 1995-June 1994), reference tracking, and consultation of colleagues  **Search strategy described:** not described in this paper, but in a previous paper [60]  **Inclusion criteria explicitly reported:** yes  **Interventions to reduce selection bias:** no  **Methodological assessment:** no  **Adequate methods used to combine studies and reach conclusions reported:** no | **Characteristics of guidelines:**  - Guidelines taking into account local circumstances are more likely to have effect  - Studies are not unanimous whether guidelines that are developed by end users (amongst others) are more used  **Characteristics of implementation strategies:**  - Passive reception of information (e.g. publication in professional journals and mailings) are usually insufficient to change behaviour  **-**  Educational interventions requiring more active participation by professionals (including targeted seminars, educational outreach visits and use of opinion leaders) are more likely to lead to changes in behaviour  - Strategies are more likely to be effective when they operate directly upon the consultation between professional and patient (e.g. restructuring medical records, patient specific reminders and patient mediated intervention)  - Educational interventions requiring active professional participation, and implementation strategies that are closely related to clinical decision making are more likely to lead to successful implementation  **-** Insufficient evidence to reach conclusions about the relative effectiveness of different strategies in different contexts.  **Professionals’ characteristics:**  - Clinicians’ concern about legal status of guidelines and potential litigation may be a barrier to implementation  **Patient characteristics:---**  **Environmental characteristics:---** | Guidelines are more likely to be effective if they take into account local circumstances, are disseminated by an active educational intervention, and implemented by patient specific reminders relating directly to professional activity. |
| **Davis & Taylor-Vaisey [57]**  **Aim:** to recommend effective strategies for implementing guidelines by clinicians  **Studies included:** English language studies that measured physicians’ performance or health care outcomes in relation to implementation strategies. Not totally clear how many studies were included (in the reference list 61 publications are mentioned).  **Guideline topics:** a variety of subjects (e.g. diabetes mellitus management, preventive strategies, smoking cessation and depression)  **Guideline target groups:** (mainly) physicians | **Design of studies included:** several designs, but particular attention was given to RCTs/ trials  **Searches in:** Medline and The Research and Development Resource Base in Continuing Medical  Education, maintained by the University of Toronto (Jan 1990 to June 1996)  **Search strategy described:** yes  **Inclusion criteria explicitly reported:** partially; for instance, unclear whether only English language publications were eligible for inclusion  **Interventions to reduce selection bias:** not mentioned  **Methodological assessment:** no  **Adequate methods used to combine studies and reach conclusions reported:** partially | **Characteristics of guidelines:**  - Guidelines that are relatively uncomplicated and could be observed or tried by the clinician are more effectively adopted  **Characteristics of implementation strategies:**  -Weak implementation interventions with scarce or no effect are didactic lecture-based continuing education (e.g., conferences and seminars) and mailed, unsolicited materials  - Moderately effective interventions concern audit and feedback, especially if done concurrently, directed at specific providers and delivered by peers or opinion leaders  - Relatively strong interventions concern reminder systems, academic detailing and multiple interventions  - Audit and feedback methods are more effective when given concurrently than when given later and retrospectively  - Strategies involving two or more interventions have more impact  **Professionals’ characteristics**  - Age and country of the (potential) users may be of influence (e.g. young Ontario medical graduates were more favourably inclined toward clinical practice guidelines than their US colleagues)  - Whether or not physicians develop their own guidelines would have no significant influence  - Factors such as physicians’ habits and customs and their reluctance to discharge patients on weekends, may be barriers  **Patient characteristics:**  - Co-morbidity negatively influences use of guidelines  - Individual demands and clinical problems (e.g. patients compliance) also affects use  - Population (demographic) perspectives may also have an effect (not further explained what is meant by this factor)  **Environmental characteristics**  - System inefficiency influence the use of guidelines  - Beliefs of peers and social norms appear to be major determinants  - Incentives related to legal or financial issues  (such as overall physician compensation or reimbursement  incentives for particular procedures) also affect the adoption of guidelines  - Regulation by accreditation or licensing bodies may affect adaptation as well | Some guideline implementation interventions are weak, others are moderately effective, while others have strong effects (also see the column to the left)  For future implementation strategies, an analysis of forces and variables influencing practice have to be made. The use of methods that are practice- and community based rather than didactic is recommended. |
| **Grilli & Lomas [53]**  **Aim**: to establish relationship  between compliance  and some key aspects  of guidelines  **Studies included:** 23 English language studies providing compliance rates with guidelines endorsed by official national organizations.  **Guideline topics:** diagnostic or treatment procedures, e.g. in cardiology, oncology, preventive medicine, dental care, obstetrics and gynaecology  **Guideline target groups:** (mainly) physicians | **Design of studies included:** quantitative designs (for studies which provided a before-after assessment only the after measurement of compliance was taken into account)  **Searches in:** Medline, reference lists of relevant reviews, bibliographies (1980 to 1991). References were also tracked down by personal contacts  **Search strategy described:** partially  **Inclusion criteria explicitly reported:** yes  **Interventions to reduce selection bias:** no  **Methodological assessment:** no  **Adequate methods used to combine studies and reach conclusions reported:** yes | **Characteristics of guidelines:**  - Target area: guidelines with recommendations on cardiovascular or cancer care had significantly higher compliance rates than those for preventive care, dental care or obstetrics and gynaecology  - Complexity (degree to which a procedure is difficult to understand or requires the availability of specific resources): highly complex recommendations had significantly lower compliance rates than those low on complexity  - Trialability (extent to which a procedure can be experimented with on a limited basis before making a final decision to adopt): highly trialable recommendations had significantly higher compliance rates  - Observability (extent to which results of a procedure are visible to those using it): no significant difference in compliance between recommendations with high versus low observability  - Type of procedure: no major difference in compliance rates emerged according to type of procedures recommended (e.g. physical examination, bioptic procedures, medical or surgical treatment)  - Elapsed time: no significant relationship was found between mean elapsed time from the release of recommendations to compliance assessment Characteristics of the implementation strategies:--- **Professionals’ characteristics:** ---  **Patient characteristics:** ---  **Environmental characteristics:** --- | Target area, complexity and trialability of recommendations appear to be predictors of compliance with guidelines |
| **Grimshaw et al. [41,42]**  **Aim: (a)** to determine and compare effectiveness and costs of different guideline dissemination and implementation strategies; (b) to estimate the resource implications of these strategies; (c) to develop a framework for deciding when it is efficient to develop and introduce clinical guidelines.  **Studies included:** 235 English language studies evaluating implementation strategies targeting medically qualified professionals, and reported objective measures of provider behaviour or patient outcomes studies.  **Guideline topics:** varying, e.g. general management of care problems, prescribing, test ordering, prevention, patient education or advise in a variety of care settings.  **Guideline target groups:** varying | **Design of** s**tudies included:** RCTs, CCTs, controlled before and after studies or interrupted time series studies  **Searches in:** Medline (1966 to 1998), HEALTHSTAR (1975 to 1998), Cochrane Controlled Trial Register (4^th^ edn 1998), EMBASE (1980 to 1998), SIGLE (1980 to 1988), specialised register of EPOC group  **Search strategy described:** yes  **Inclusion criteria explicitly reported:** yes  **Interventions to reduce selection bias:** 2 reviewers screened the search results, but it is not reported whether they did this independently  **Methodological assessment:** 2 reviewers independently abstracted data on methodological quality using the EPOC group’s methodological criteria  **Adequate methods used to combine studies and reach conclusions reported:** yes | **Characteristics of guidelines:---**  **Characteristics of implementation strategies:**  *Multi-faceted strategies:*  - Multi-faceted strategies including educational outreach may have a modest effect on guideline implementation, especially when targeting prescribing behaviours  - Educational materials and educational meetings in combination may have, at best, a small effect on guideline implementation.  - Educational materials, educational meetings, and audit and feedback in combination may have, at best, a small effect on guideline implementation  - Combinations of reminders and patient-directed interventions may lead to moderate effects  - Educational materials, educational meetings and organisational interventions in combination may have, at best, a small effect on guideline implementation  - Educational outreach appeared to be more effective than educational materials  - The combination of educational materials and reminders appears more effective than educational materials alone  - The combination of educational materials, educational meetings and reminders appears more effective than educational materials and educational meetings alone  *Single strategies:*  - Educational materials may have a modest effect on guideline implementation However, the evidence base is sparse and of poor quality  - There are relatively few evaluations of educational meetings against a no intervention control. The results suggest that the effects, if any, are likely to be small  - Audit and feedback may have a modest effect on guideline implementation  - Patient mediated interventions, in the sense of new clinical information collected directly from patients and given to the provider, may result in moderate to large improvements in performance, especially when targeting preventive services  - Reminders may have a moderate effect on guideline implementation  *Comparison multi-faceted strategies and single strategies:*  - Multi-faceted interventions do not appear to be more effective than single interventions  - No relationship was found between number of components of multi-faceted strategies and the effects measured  **Professionals’ characteristics:** ---  **Patient characteristics:** ---  **Environmental characteristics:** --- | The overall quality of the included studies was poor. There is an imperfect evidence base to support decisions about which guideline dissemination and implementation strategies are likely to be efficient under certain circumstances |
| **Gross & Pujat [54]**  **Aim:** to determine which guideline implementation methods improve outcomes of appropriate antimicrobial use  **Studies included:** 40 English language studies concerning effects of guideline dissemination and implementation strategies.  **Guideline topics:** antimicrobial use in common infections (e.g. otitis media, urinary and surgical wound infections)  **Guideline target groups:** physicians and sometimes also others ( nurses, pharmacists) | **Design of studies included:** (RCTs, CCTs, before-and-after studies  **Searches in:** Medline, Cochrane database (to December 2000)  **Search strategy described:** partially  **Inclusion criteria explicitly reported:** yes  **Interventions to reduce selection bias:** no  **Methodological assessment:** no  **Adequate methods used to combine studies and reach conclusions reported:** partially | **Characteristics of guidelines:---**  **Characteristics of implementation strategies:**  - Most successful strategies (often combined): academic detailing, local adaptation of the guideline, small-group sessions, written feedback and computer-assisted care  - Multi-faceted interventions are more likely to succeed, although it is unclear which components account for the success  - Multi-faceted educational interventions are particularly successful when they have an interactive component  - Educational brochures or didactic education programs alone seems to be not effective  - Dissemination of guidelines appears to be useful only when accompanied by extensive media support and other educational methods  - Computer-assisted implementation methods appear to be effective when attached to computer-based patient records and laboratory record and when the physician enters the orders on the computer  - Early involvement of relevant non physician health care providers (e.g. nurses and pharmacists) facilitates guideline implementation.  **Professionals’ characteristics:** ---  **Patient characteristics:** ---  **Environmental characteristics:** --- | It was not possible to distinguish the effectiveness of the guideline from the effectiveness of the implementation methods Although some single implementation methods appear to be also useful, multi-faceted implementation methods are most successful |
| **Sachs [51]**  **Aim :** to get evidence on factors influencing the implementation of guidelines into nursing practice  **Studies included:** 15 English- or German language studies on local, regional or national guidelines, focussing on 1 or more implementation strategies.  **Guideline topics:** varying, e.g. pre- and postoperative care, dermatological care, pressure ulcers, oral care, et cetera.  **Guideline target groups:** nurses, sometimes combined with other physicians or other professionals | **Design of studies included:** RCTs, CCTs, pre-test post-test and post-test only designs, and 1 systematic review  **Searches in:** Medline, Cochrane Library and some German language literature catalogues, combined with reference tracking. Search period not mentioned  **Search strategy described:** yes  **Inclusion criteria explicitly reported:** yes  **Interventions to reduce selection bias:** no  **Methodological assessment:** no  **Adequate methods used to combine studies and reach conclusions reported:** partially | Characteristics of guidelines: - When the guideline is developed by the target group and experts (sometimes in addition to other persons) this enhances the chance of successful implementation  **Characteristics of implementation strategies:**  - Systematic analysis of the situation in practice before the implementation, improves chance of successful implementation  - Educational interventions and active implementation strategies (e.g. giving the professionals involved additional tasks, involving experts in the implementation process) have a larger effect than only passive dissemination of guidelines  - Multiple strategies in which, for instance, individual instructions, feedback and reminders are combined, are more successful than separate strategies  - Multiple materials or strategies (e.g. practical recommendations, written material, educational meeting and supervision) improves the chance of implementation success  - Educational interventions using active and problem oriented methods, in which attention is being paid to the specific target group and the practice setting are often successful Professionals’ characteristics: - limited motivation has a negative influence on the success of implementation  - Openness to innovations and previous experiences with innovations have a positive influence  - (Un)clear expectations also have influence  **Patient characteristics:** ---  **Environmental characteristics:**  - Support of managers influences implementation. Support may include, for instance, financial support to create possibilities to participate in educational meetings, to arrange the needed material or aids, and active involvement of superiors in the implementation process  - Limited time or personnel, many changes and instability in the organization, all have negative influence | There are indications that effective strategies to implement nursing guidelines concern active involvement of practitioners and are practically orientated. Multi-faceted strategies addressing both the introduction process and organisational conditions appear to be most effective.  Passive distribution of nursing guidelines reveals little success. |
| **Saillour-Glenisson & Michel [52]**  **Aim :** to study facilitators and barriers to physicians’ adherence to guidelines  **Studies included: 59** English or French language studies on facilitators or barriers to physicians’ adherence to guidelines**.**  **Guideline topics:** varying, e.g.. cancer treatments, care for chronic patients, treatment of high blood pressure, alcohol addiction and several preventive and diagnostic interventions.  **Guideline target groups:** (mainly) physicians | **Design of studies included:** several quantitative and qualitative designs or combined qualitative/quantitative designs.  **Searches in:** Medline, Current contents, Cochrane Library, Healthstar (1966 to 2001). References were also tracked down by studying bibliographies and consultation of with experts  **Search strategy described:** yes  **Criteria for inclusion:** yes  **Interventions to reduce selection bias:** no  **Methodological assessment:** no  **Adequate methods used to combine studies and reach conclusions reported:** partially | Characteristics of guidelines: - Form and subject of guidelines influence physicians’ adherence to guidelines (e.g. relevance of the subject, concretely and clearly stated)  - Applicability (e.g. regarding the complexity, compatibility with already existing procedures/practices, and the costs of executing recommendations) also has influence  - Scientific basis: adherence of evidence based guidelines is higher  - Observability; the extent to which clinical benefits of the guideline are visible  - Trialability: the extent in which recommended procedures can be tried out has also influence  - Adaptability; when adapting the guideline to local circumstances the adherence is higher  - Legal implications of adhering recommendations are also influential  **Characteristics of implementation strategies:---** Professionals’ characteristics: - Knowledge and attitude: the extent in which physicians know the content of the guideline is a determinant, as well as their attitude and agreement to the guideline  - Psychological characteristics (e.g., self efficacy), age and other socio demographic characteristics), income, training and job satisfaction are also influential  **Patient characteristics:**  - Patient characteristics (e.g. educational level), patients’ attitude (e.g. resistance) and patient-doctor interactions also influence physicians’ adherence  **Environmental characteristics:**  - Structural characteristics of the work environment (e.g. working in rural areas or not, working in day or night shifts etc., working in close collaboration with other physicians) also influences adherence  - Financial and reimbursement characteristics: financial incentives influence adherence as well  - Availability of relevant materials and aids also have influence  - Work characteristics such as flexibility of the work and work pressure  - Interactions with and attitudes of colleagues are influential as well  - Sufficient material and physician internal organizational environment also influence adherence | There are many factors influencing adherence. Interpretation of results is hampered by absence of conceptual frameworks by absence of multi-factorial analysis. |
| **Simpson et al. [50]**  **Aim:** (a) to evaluate effects of controlled studies of guideline-based interventions on processes and outcomes of care for community-acquired pneumonia; (b) to identify barriers to the adoption and use of guidelines. (Only aim b is relevant for our meta-review)  **Studies included:** English language studies: 6 evaluated the effectiveness of guidelines and 8 described barriers to guideline adoption and use  **Guideline topics:** community-acquired pneumonia  **Guideline target groups:** (mainly)  physicians | **Design of studies included:** with regard to aim (a) only before-after studies with concurrent external controls, time series analyses or RCTs. With regard to aim (b) several kinds of designs (e.g. surveys) were included.  **Searches in:** Medline (to July 2004). References were also tracked down by studying bibliographies of articles and consultation of experts.  **Search strategy described:** yes  **Inclusion criteria explicitly reported:** yes  **Interventions to reduce selection bias:** no  **Methodological assessment:** no  **Adequate methods used to combine studies and reach conclusions reported:** partially | Characteristics of guidelines: - Complexity, user-unfriendliness and limited accessibility of guidelines may limit use of pneumonia guidelines.  - Discordance among various sets of guidelines is also a barrier  - Lack of local ownership is also a barrier  **Characteristics of implementation strategies:** - Ongoing support in the form of reminders (pre-printed orders or computer decision tool) and dedicated nurses or other allied health professionals operationalizing the recommendations help physicians to overcome barriers for use  **Professionals’ characteristics:**  - Lack of physicians’ awareness or agreement with pneumonia guidelines are a barrier for adoption  - Physicians’ conservative attitudes also form a barrier  - Physicians less experienced are more likely to follow guidelines  - Legal concerns influence whether or not guidelines are followed  **Patient characteristics:**  - Age: older patients (65+) are less likely to be managed according to pneumonia guidelines  - Severe pneumonia and co-morbidities are also barriers - Non-clinical patient factors (e.g. patient demands or expectations, presence of social support, insurance status, reliability, adequacy of outpatient follow-up) also influence physicians’ adherence  **Environmental characteristics:**  - Limited time, personnel and resources devoted to support guideline adherence, and high workload are barriers in guideline adherence | There are many barriers at the level of the patient, the physician, and the health care system to the adoption and use of guidelines on community-acquired pneumonia. |
| **Thomas et al. [56]**  **Aim:** to identify and assess the effects of studies of the introduction of guidelines in nursing,  midwifery or other professions allied to medicine.  **Studies included:** 18 English language studies, of which only 3 were comparing different dissemination and/or implementation strategies (which is relevant for our review).  **Guideline topics**: varying, e..g. urinary care, management of hypertension, low back pain, nutrition therapy etc.  **Guideline target groups:** nurses, midwifes or other allied health professionals. | **Design of studies included:** RCTs, CCTs, before-and-after studies or interrupted time series  **Searches in:** Medline, Cinahl, Cochrane EPOC Group specialised register, DARE , DHSS-Data, EMBASE, NHS Economic Evaluations Database and Sigle, (all to 1996) and also by studying reference lists, consultation of experts and hand searches.  **Search strategy described:** yes  **Inclusion criteria explicitly reported:** yes  **Adequate methods used to combine studies and reach conclusions reported:** partially  **Interventions to reduce selection bias:** two reviewers independently assessed for inclusions in the review  **Methodological assessment:** two reviewers independently assessed study quality  **Adequate methods used to combine studies and reach conclusions reported:** partially | **Characteristics of guidelines:---**  **Characteristics of implementation strategies:**  Three studies included in this review compared the effectiveness of two or more implementation strategies (e.g. a combination of receiving a guideline, lectures and opinion leaders, compared to a combination of receiving a guideline and opinion leaders and another combination of receiving the guideline and lectures). Thomas et al. did not present conclusions in this regard, since the three relevant studies were compromised by a small sample size or unit of analysis errors.  **Professionals’ characteristics:** ---  **Patient characteristics:** ---  **Environmental characteristics:** --- | There is insufficient evidence to determine the effectiveness of different dissemination and implementation  strategies. More research is needed to determine the effectiveness of different strategies. |
| **Tooher et al. [55]**  **Aim:** to explore how pressure ulcer guidelines best can be implemented.  **Studies included:** 20 studies on the effects of various implementation strategies on outcomes of care, processes of care, processes of change or resource use or costs.  **Guideline topic:** Pressure ulcers treatment  **Guideline target groups:**  nurses and often also other health professionals (e.g. physiotherapists) or policy makers | **Design of studies included:** RCTs, CCTs, case series and care reports.  **Searches in:** Ovid PreMedline and Medline, Current Contents, Cochrane Controlled Trials Register and Database of Systematic Reviews; DARE; Embase, Health Technology Assessment Database, NHS Economic Evaluation Database; UK National Research register, National Inst. of Health Clinical Trials Database (to 2002).  **Search strategy described:** yes (on www.nicls.com.au)  **Inclusion criteria explicitly reported:** yes  **Interventions to reduce selection bias:** no  **Methodological assessment:** no  **Adequate methods used to combine studies and reach conclusions reported:** partially | Characteristics of guidelines:--- **Characteristics of implementation strategies:** - Providing targeted information or educational sessions, documentation aids, institutional and management support, as well as active monitoring of adherence to guidelines appears to be successful. - Only distributing guidelines without education support is less successful. - Use of computer-based decision aids does not affect decision-making regarding pressure ulcer care.  - Comprehensive pressure ulcer programmes involving multi-faceted implementation strategies across the institution appear to be also effective in the long-term.  **Professionals’ characteristics:** ---  **Patient characteristics:** ---  **Environmental characteristics:** --- | Active strategies are associated with better outcomes than dissemination only. Targeted educational sessions appear to be common to studies reporting positive outcomes after implementation.  The more comprehensive implementation strategies are, the more effective the implementation. |
